# Supplementary material for: Time-Course of Transcriptomic Change in the Lungs of F344 Rats Repeatedly Exposed to a Multiwalled Carbon Nanotube in a 2-Year Test
Source: Nanomaterials (Basel). 2023 Jul 19;13(14):2105. doi: 10.3390/nano13142105 (PMC10383707; doi:10.3390/nano13142105)
Supplement: Supplementary file 1 [file nanomaterials-13-02105-s001.zip › Supplementary Figures Hojo Nanomaterials_revised MH revised_July 19.pdf]

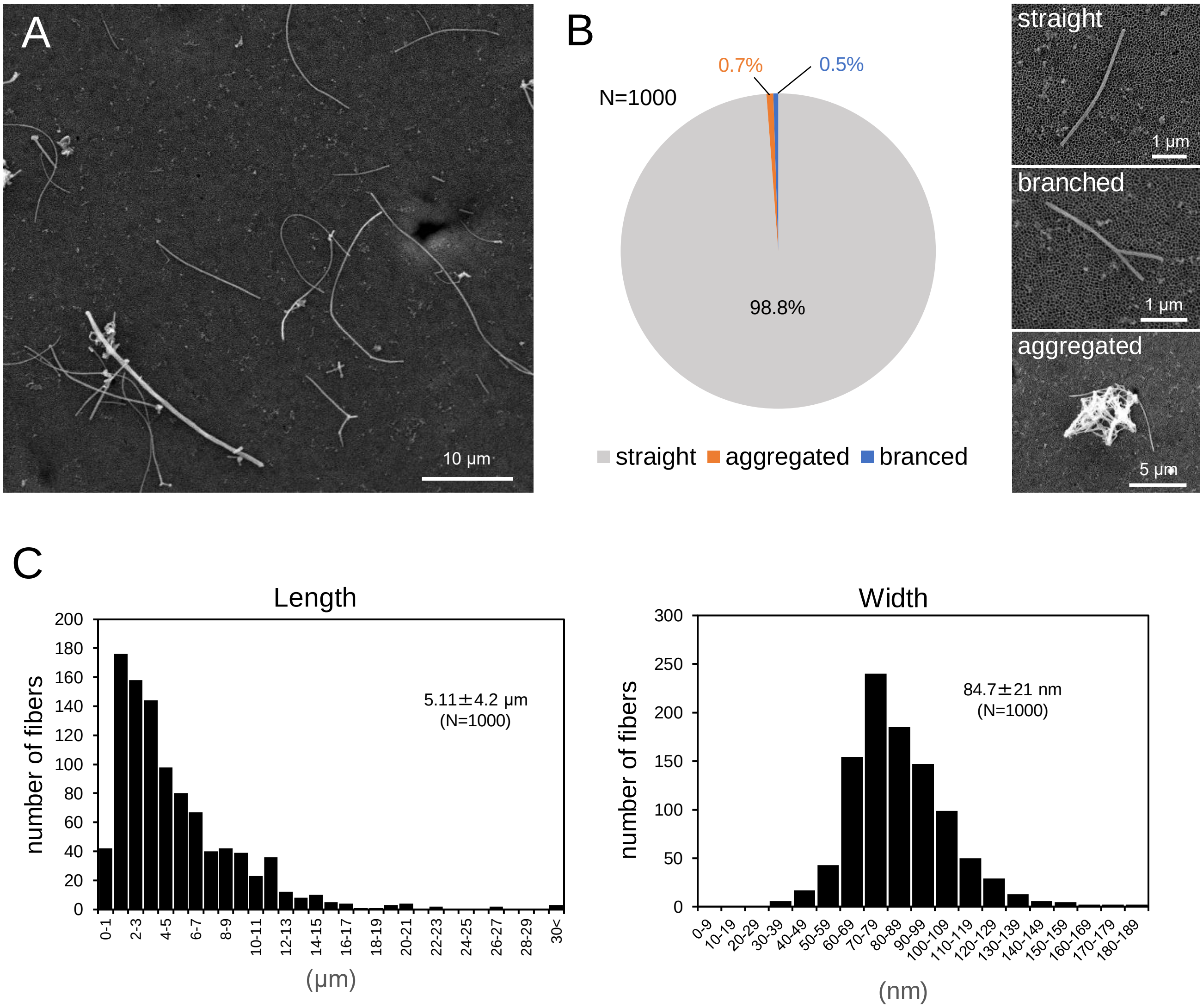

Figure S1. Characterization of the test material using a scanning electron microscope (Quanta™ FEG250; Thermo Fisher Scientific).

(A) SEM image of Taquann treated-MWNT-7. (B) Morphological classification of the individual fibers. The straight fibers account for nearly 99% of all examined fibers (left). Representative images of the 3 structures (right). (C) Length and width distribution of MWNT-7.

Detailed data were described in previous study by Hojo *et al.* (https://doi.org/10.1186/s12989-022-00478-7)

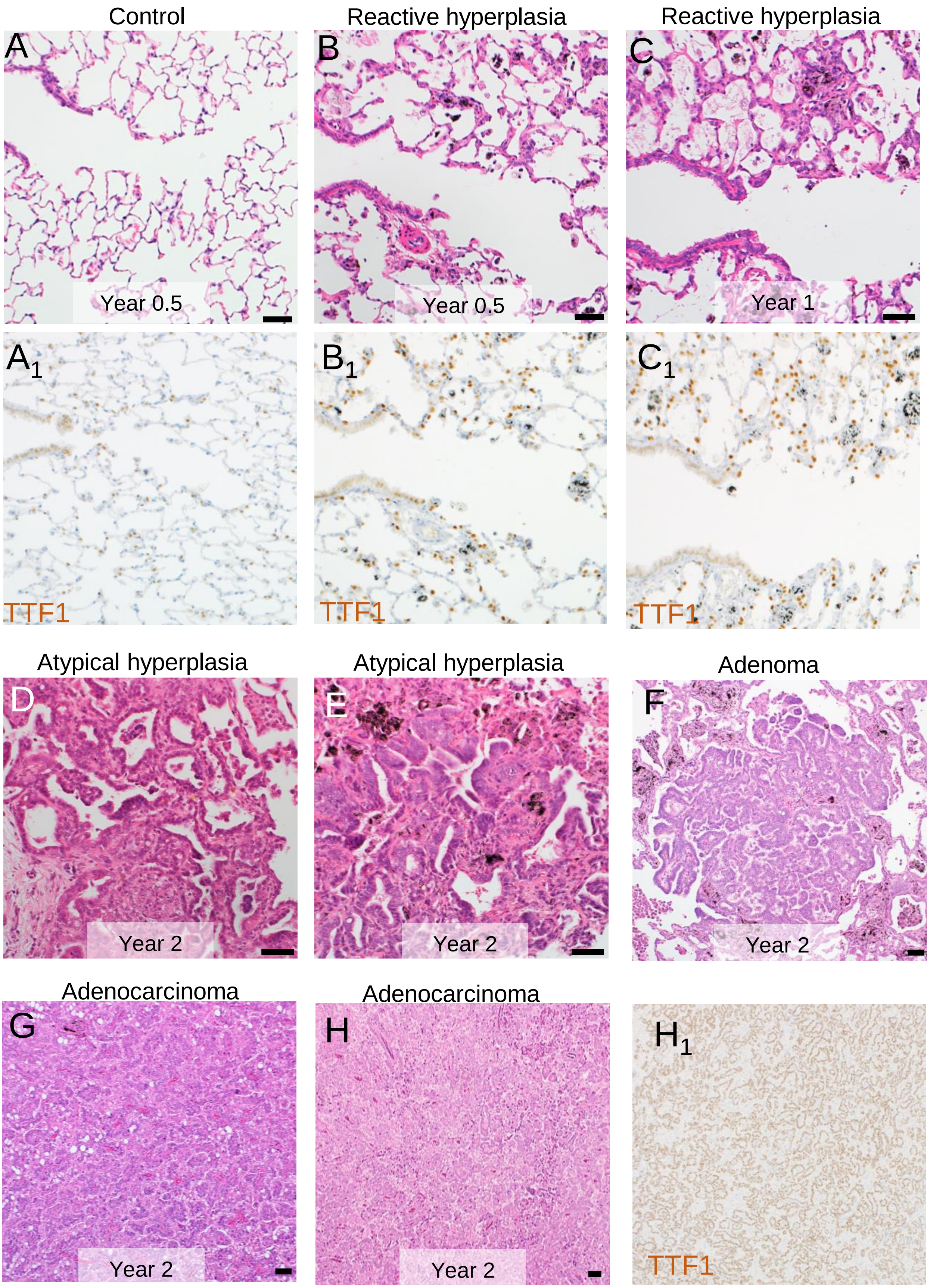

Figure S2. Representative histological images of proliferative lesions in the lung of MWNT-7-treated rats in the main 2-year study

(A)–(C) Region around the terminal bronchiole of the lung in rats of the control group (A), MWCNT-treated group at Year 0.5 (B), and MWCNT-treated group at Year 1 (C). Foci of reactive alveolar hyperplasia are scattered. Immunostaining for TTF-1 in serial sections of A, B, and C (A1, B1, and C1). The proliferation of the alveolar epithelium is evidenced by an increase in the immunopositivity of the nuclei for TTF-1 in the MWNT-7 treated group. (D) and (E) Atypical hyperplasias. Irregular structures, such as partially multilayered epithelia, protrusion of clustered cells, and glandular structures, are seen with fibrously thickened septa. (F)– (H) Lung tumors found in MWNT-7-treated rats. (F) Bronchiolo-alveolar adenoma. Underlying alveolar architecture is obscure, and the tumor mass appears to compress the surrounding tissue. (G) and (H) Bronchiolo-alveolar adenocarcinomas. The tumor exhibits glandular and papillary growth patterns with structural atypia. (H1) Immunostaining for TTF-1 in a serial section of H. Scale bar: 50 µm.

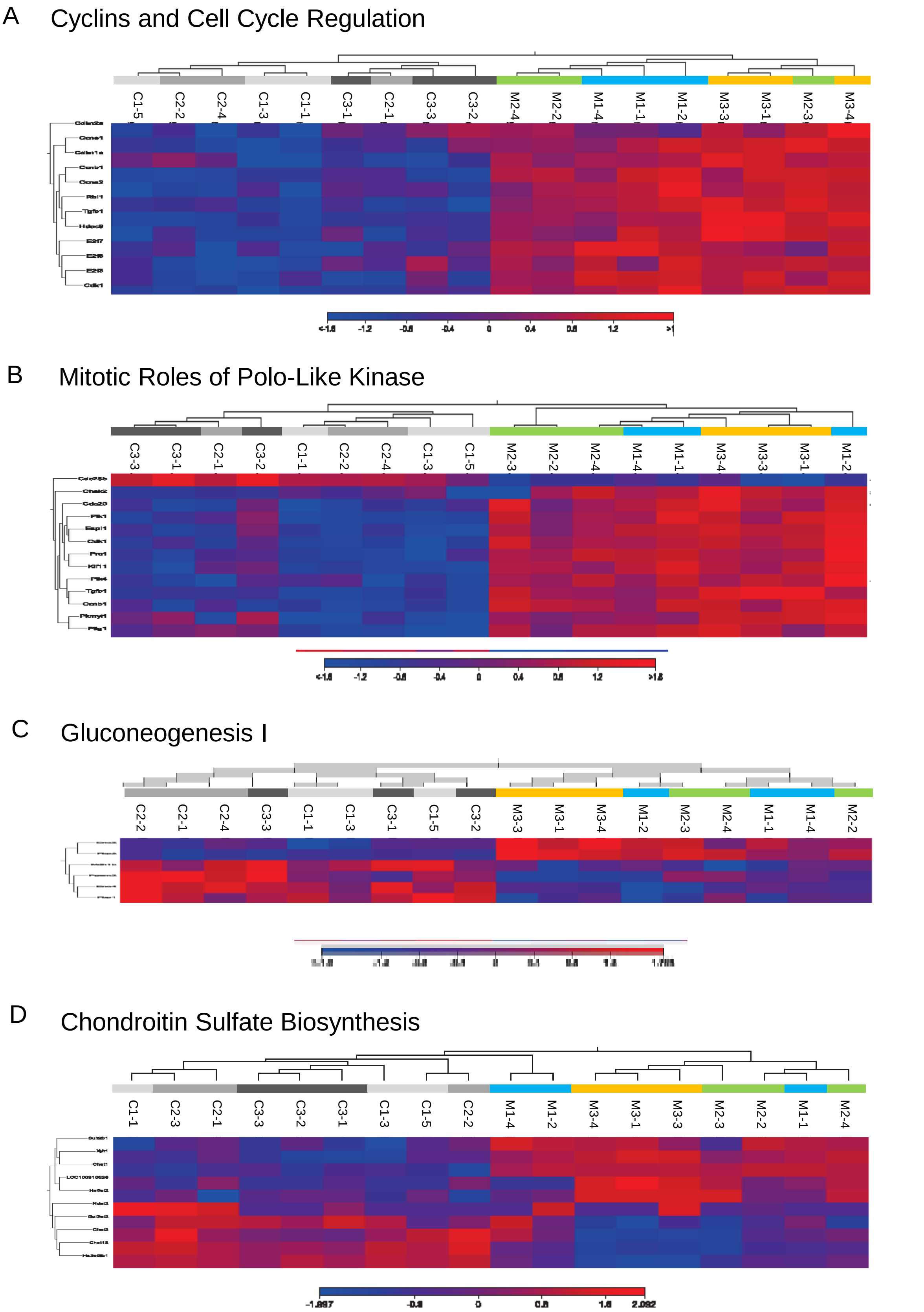

Figure S3. Gene expression changes in pathways perturbed at Year 0.5 or Year 1
Heatmaps resulting from hierarchical clustering of DEGs belonging to the pathway. (A) Cyclins and Cell Cycle Regulation. (B) Mitotic Roles of Polo-Like Kinase. (C) Gluconeogenesis I. (D) Chondroitin Sulfate Biosynthesis. Color bar indicates high-expressed (red) and low-expressed (blue) genes. N = 3 per time point.

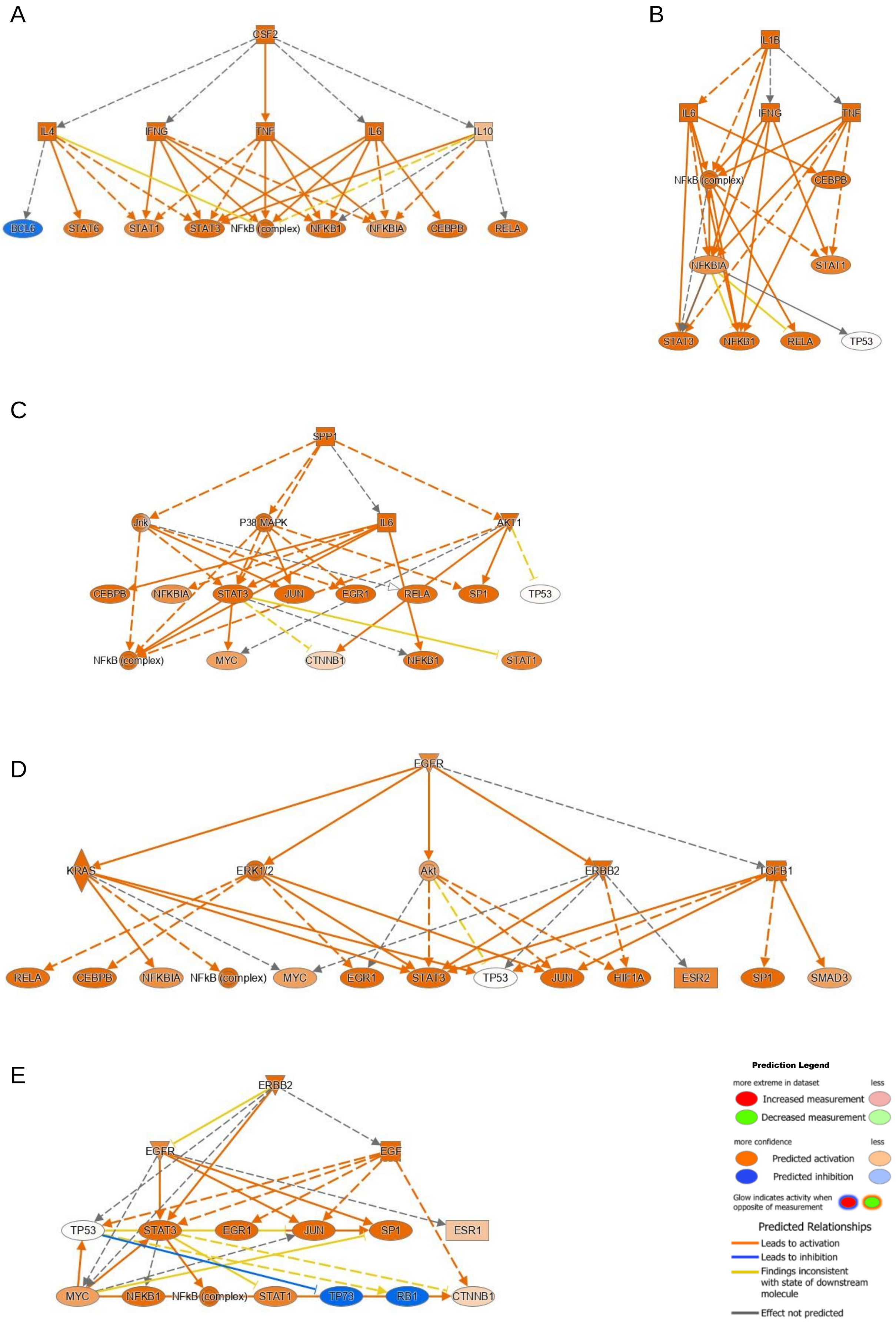

Figure S4. Examples of Upstream Regulators and Mechanistic Networks

Upstream Regulators and their predicted targets are shown as mechanistic networks. (A) Colony Stimulating Factor 2 (CSF2). (B) Interleukin 1b (IL1B). (C) Secreted Phosphoprotein 1/Osteopontin (SPP1). (D) Epidermal Growth Factor Receptor (EGFR). (E) Erb-B2 Receptor Tyrosine Kinase 2/Her2/Neu (ERBB2). Detailed descriptions of the IPA results can be found in the QIAGEN website (https://qiagen.my.salesforce-sites.com/KnowledgeBase/KnowledgeNavigatorPage?categoryName=IPA).

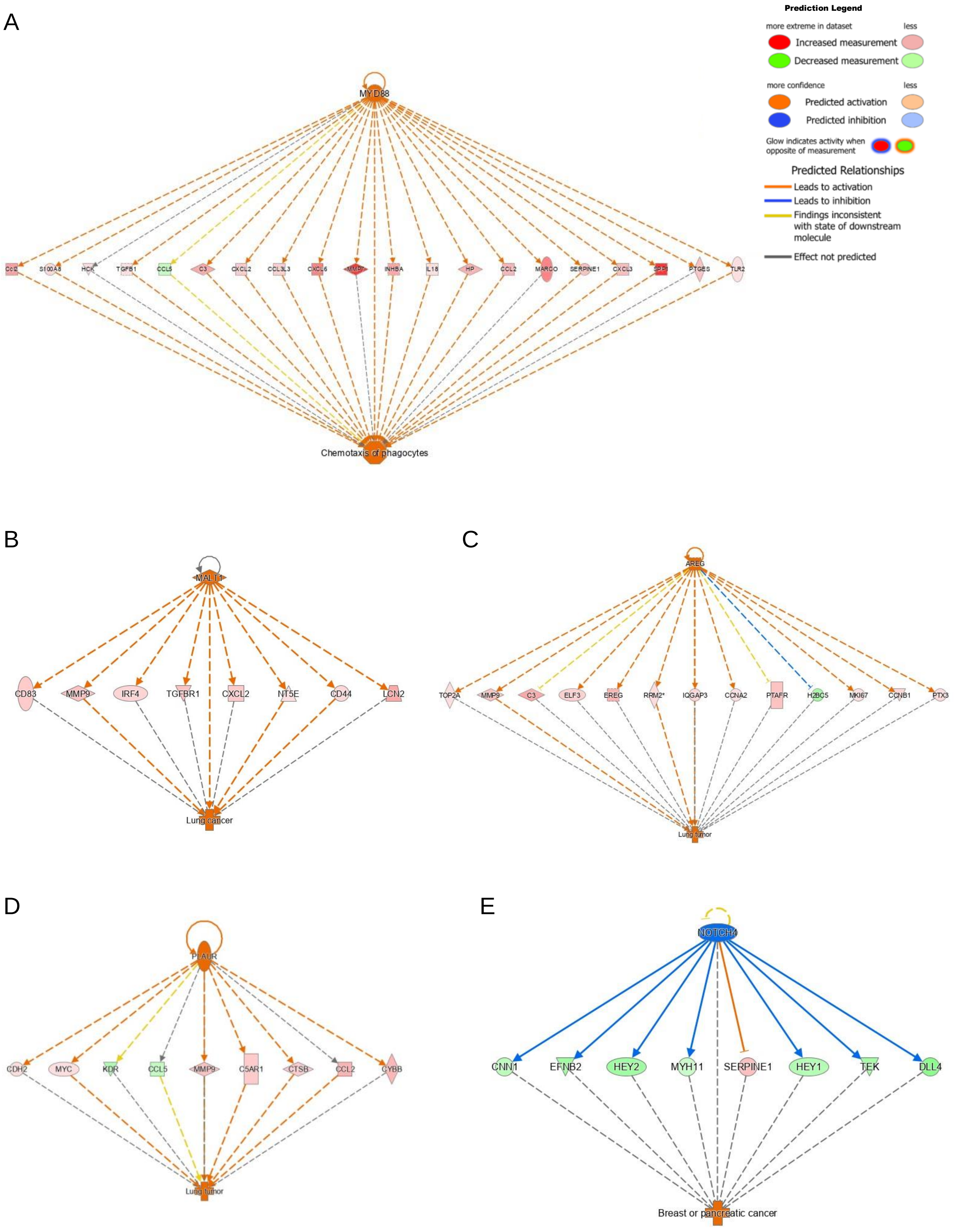

Figure S5. Examples of Regulator Effects

Five selected Regulator Effects are shown. (A) Myeloid Differentiation Primary Response Protein MyD88 (MYD88). (B) MALT1 Paracaspase (MALT1). (C) Amphiregulin (AREG). (D) Urokinase Plasminogen Activator Surface Receptor (PLAUR). (E) Notch4. Detailed descriptions of the IPA results can be found in the QIAGEN website (https://qiagen.my.salesforce-sites.com/KnowledgeBase/KnowledgeNavigatorPage?categoryName=IPA).

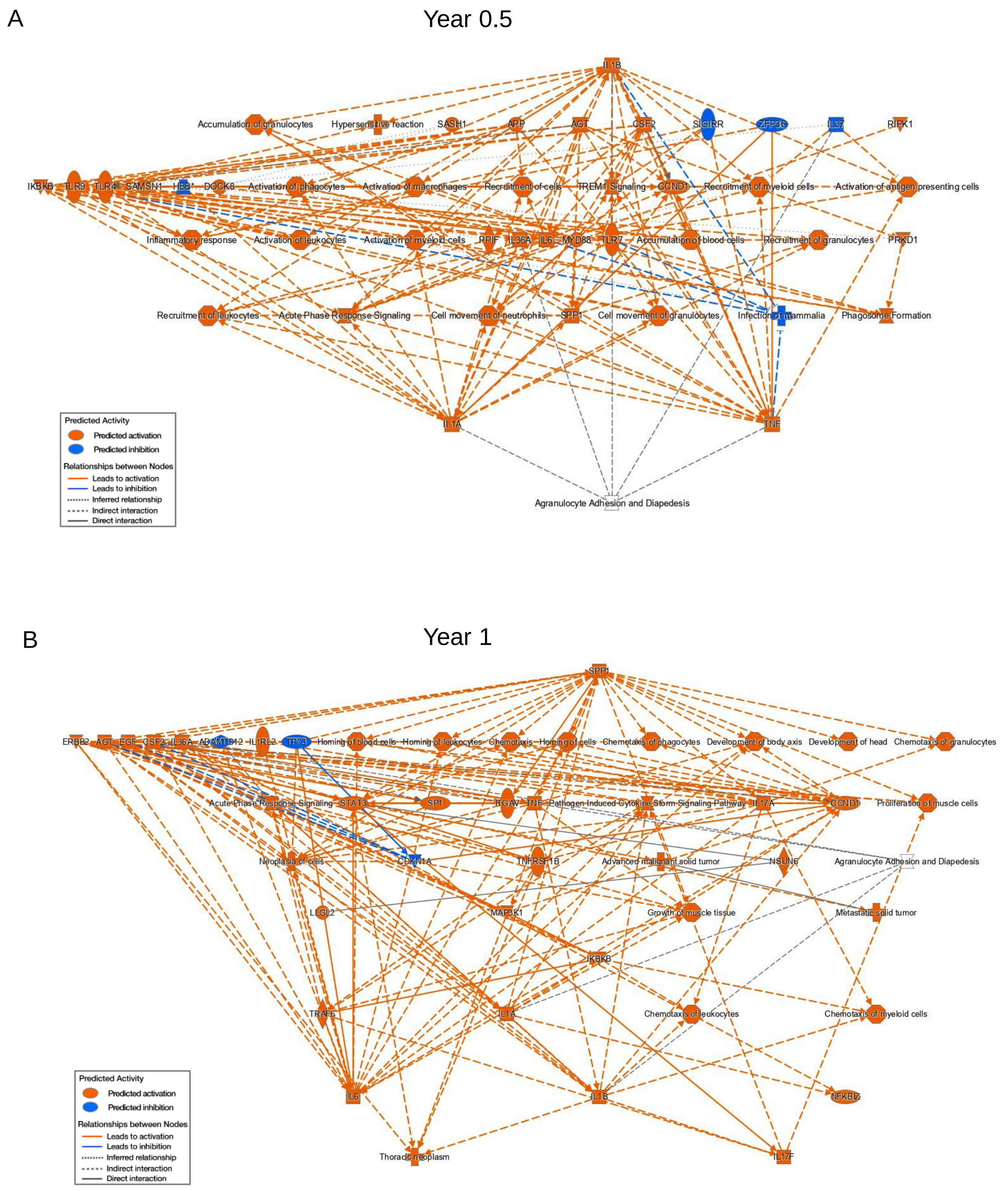

Figure S6. Graphical Summary of the Core Analysis of each time point

C

Year 2

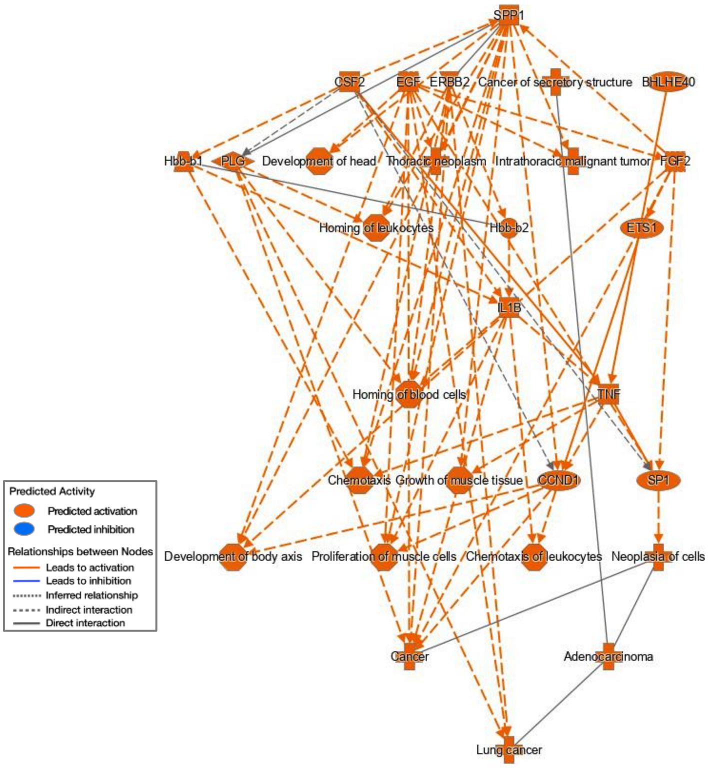

Figure S6. Graphical Summary of the Core Analysis of each time point (continued)

(A) Graphical Summary based on the data set of Year 0.5. (B) Graphical Summary based on the data set of Year 1. (C) Graphical Summary based on the data set of Year 2 (see Materials and Methods). Means of node shapes and other details of the graphics can be found in the QIAGEN website (https://qiagen.my.salesforce-sites.com/KnowledgeBase/KnowledgeNavigatorPage?categoryName=IPA).

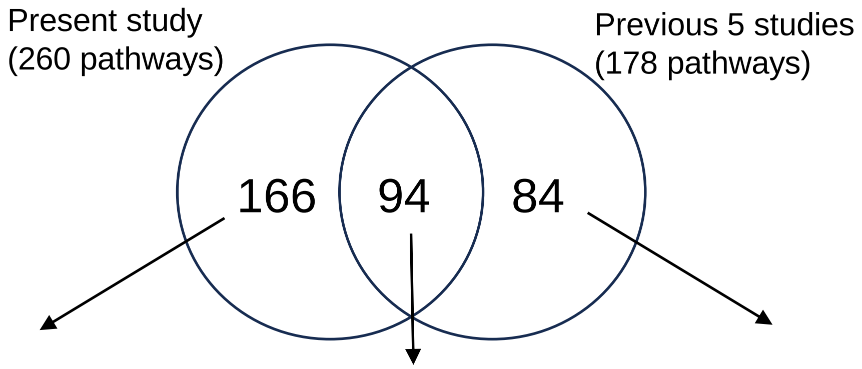

14-3-3-mediated Signaling	Acute Phase Response Signaling	B Cell Activating Factor Signaling
Apelin Cardiomyocyte Signaling Pathway	Agranulocyte Adhesion and Diapedesis	Calcium-induced T lymphocyte apoptosis
Axonal Guidance Signaling	Antioxidant Action of Vitamin C	Cell Cycle Control of Chromosomal Replication
CDK5 Signaling	cAMP-mediated signaling	IGF-1 Signaling
Ephrin B Signaling	Coagulation System	LPS-stimulated MAPK Signaling
Ephrin Receptor Signaling	Colorectal Cancer Metastasis Signaling	Nicotine Degradation II
ERBB Signaling	Complement System	Phospholipase C signaling
ERK/MAPK Signaling	FXR/RXR Activation	PPAR Signaling
FAK Signaling	G-Protein Coupled Receptor Signaling	Pyrimidine Ribonucleotides De Novo Biosynthesis
Gluconeogenesis I	Granulocyte Adhesion and Diapedesis	Retinol Biosynthesis
Mitotic Roles of Polo-Like Kinase	Hepatic Fibrosis / Hepatic Stellate Cell Activation	Th17 Activation Pathway
MSP-RON Signaling In Cancer Cells Pathway	Kinetochore Metaphase Signaling Pathway	Xenobiotic Metabolism AHR Signaling Pathway
Netrin Signaling	LXR/RXR Activation	Xenobiotic Metabolism CAR Signaling Pathway
Notch Signaling	p38 MAPK Signaling	
P2Y Purigenic Receptor Signaling Pathway	Phagosome Formation	
Phospholipases	Phagosome Maturation	
Role of MAPK Signaling in Promoting the Pathogenesis of Influenza	Th1 and Th2 Activation Pathway	
Semaphorin Neuronal Repulsive Signaling Pathway	Toll-like Receptor Signaling	
Sonic Hedgehog Signaling	TREM1 Signaling	
	Tumor Microenvironment Pathway	

Figure S7. Comparison of canonical pathways between the present study and previous 5 studies.
Venn diagram: Left circle shows all significantly perturbed pathways in this study (260; after removing redundant pathways among 3 time points). Right circle shows all listed perturbed pathways in previous studies (178; after removing redundant pathways). They are extracted from 5 studies employing IPA for data analysis: Poulsen *et al.*, 2013; Poulsen *et al*., 2015; Khaliullin *et al.,* 2020; Sager *et al.,* 2022; and Solorio-Rodriguez *et al.*, 2023. Details of each study are summarized in Tables S8 and S9.
Table: Examples of pathways contained in each compartment.
